# Primary reconstruction of total maxillectomy defect with additively manufactured subperiosteal implant and fibula free flap: a case report

**DOI:** 10.1007/s10006-025-01379-7

**Published:** 2025-04-21

**Authors:** Giacomo De Riu, Andrea Biglio, Alessandro Baj, Antonino Maniaci, Jerome R. Lechien, Luigi Angelo Vaira

**Affiliations:** 1 Maxillofacial Surgery Operative Unit, Department of Medicine, Surgery and Pharmacy, University of Sassari, Viale San Pietro 43B, Sassari, Italy; 2 Maxillofacial Surgery Operative Unit, University of Milan, Milan, Italy; 3 Department of Medicine and Surgery, University of Enna Kore, Enna, Italy; 4 Department of Surgery, Mons School of Medicine, UMONS Research Institute for Health Sciences and Technology, University of Mons (UMons), Mons, Belgium; 5 Department of Otolaryngology-Head Neck Surgery, Elsan Hospital, Paris, France

**Keywords:** Maxillectomy, Maxillary reconstruction, Subperiosteal implantation, Custom made implant, Dental implant, Maxillofacial surgery

## Abstract

**Background:**

Total maxillectomy for malignant tumors presents significant challenges for functional and aesthetic rehabilitation. Advances in digital planning and additive manufacturing have reintroduced subperiosteal implants as a promising solution for primary reconstructions.

**Case report:**

This report details the case of a 59-year-old male with squamous cell carcinoma of the hard palate extending contralaterally, treated with a total maxillectomy and primary reconstruction using an osteomyocutaneous fibula free flap combined with a custom-made, additively manufactured subperiosteal implant. Preoperative planning integrated craniofacial CT scans, dental impressions, and lower limb angiography to design both the implant and fibula cutting guides. The implant, fabricated via direct metal laser sintering, provided a stable framework for fibular segments and future prosthetic rehabilitation. At two years post-surgery, the patient remains disease-free, with no complications and fully functional prosthetic integration.

**Conclusions:**

This case highlights the feasibility, adaptability, and potential benefits of additively manufactured implants in reconstructing total maxillectomy defects.

**Clinical trial number:**

N/A

## Introduction

Maxillectomy procedures, performed to resect malignant or otherwise extensive lesions of the maxilla, often entail profound alterations in the patient’s functional status and overall quality of life. Alongside the inevitable facial disfigurement, patients frequently endure compromised mastication, speech articulation difficulties, and airway management challenges [1, 2]. These functional deficits, coupled with altered facial aesthetics and potentially impaired psychosocial well-being, highlight the importance of restoring or approximating preoperative conditions as closely as possible [3].

Consequently, reconstructing maxillary defects represents a complex but crucial endeavor. The ideal reconstruction must re-establish anatomical contours, recreate a functional separation between oral and nasal cavities, restore masticatory capabilities, and allow for intelligible speech [4]. This multifaceted challenge grows even more complicated when considering the rehabilitation of dental function. Traditional approaches using osteointegrated implants may be hampered by inadequate bone volume, poor-quality soft tissues, and delicate vascularized free flaps, often making implant-prosthetic rehabilitation both technically demanding and unpredictable [5].

In recent years, however, advances in digital planning, computer-aided design, and additive manufacturing have revitalized interest in subperiosteal implants. Once overshadowed by the rise of conventional endosseous implants, subperiosteal devices have re-emerged, especially for full-arch rehabilitations in severely atrophic jaws [6–9]. Their custom, patient-specific design and the precision offered by metal 3D printing processes have allowed for improved fit, stability, and prosthetic outcomes. Following their success in secondary maxillary reconstructions [10–13], where the existing anatomy can be assessed long after resection, a few authors have contemplated their application in primary reconstructions. Yet, this approach is still in its infancy, with only a single previous report documenting the feasibility of employing custom-made subperiosteal implants at the time of initial maxillary reconstruction [14]. Concerns regarding intraoperative anatomical unpredictability, evolving postoperative tissue conditions, and the logistical complexities of producing a custom implant in a short timeframe have likely limited their broader adoption in primary cases [11, 12].

In this case report, we present a patient who underwent a total maxillectomy for squamous cell carcinoma and received a primary reconstruction incorporating a custom-designed, additively manufactured subperiosteal implant combined with an osteomyocutaneous fibular flap.

## Case report

**Fig. 1:**
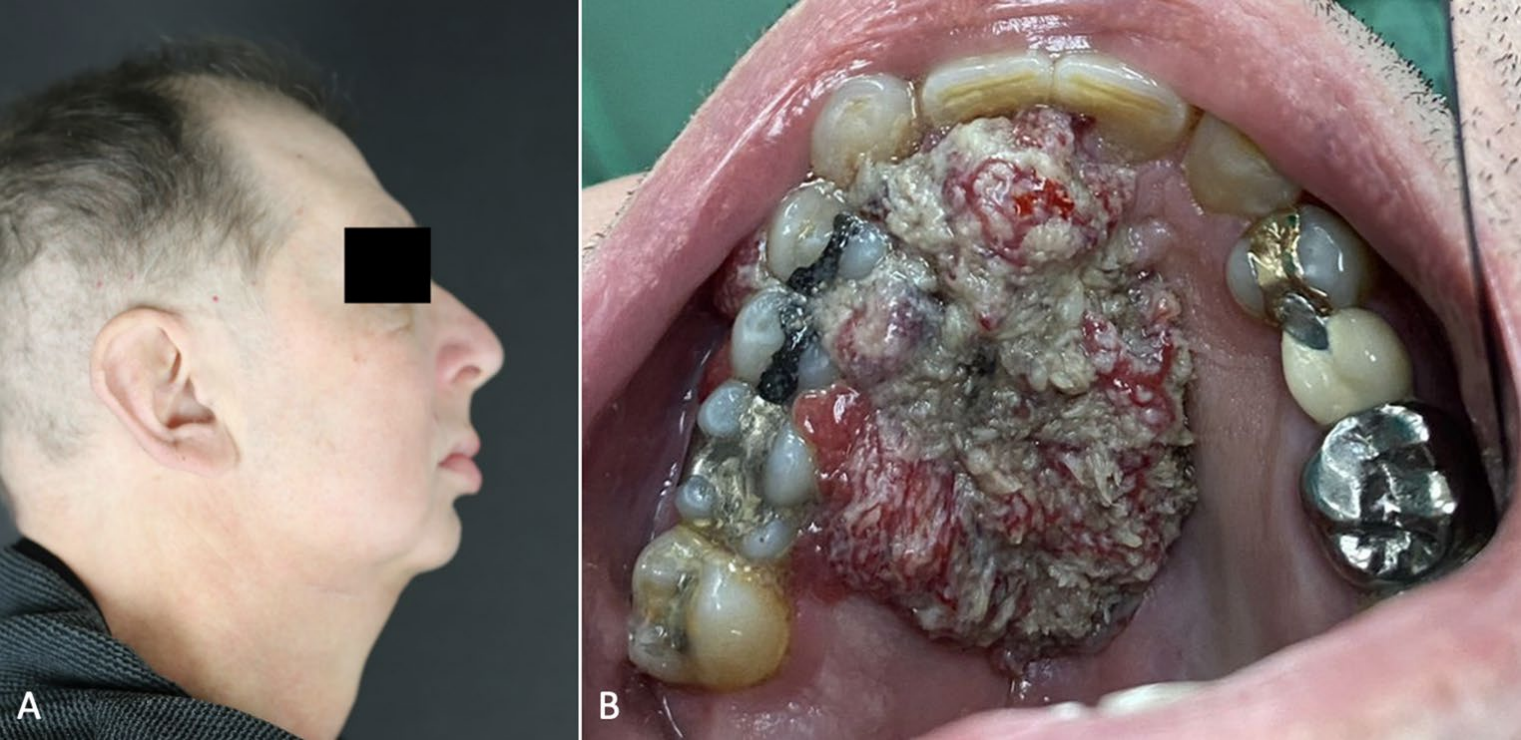
Preoperative presentation. (**A**) Lateral facial profile of the patient. (**B**) Intraoral view revealing the squamous cell carcinoma lesion involving the hard palate and extending contralaterally

A 58-year-old male patient presented to the Maxillofacial Surgery Department of the University Hospital of Sassari, Italy, with a left-sided hard palate squamous cell carcinoma extending to the contralateral side (Fig. 1). The participant provided informed consent for the use and publication of their data and image in this manuscript, with a signed consent form.

Preoperative clinical examination and imaging studies, including contrast-enhanced CT and MRI, were performed to define the local tumor extent and evaluate for regional or distant metastases. Following the multidisciplinary tumor board review, the patient was scheduled for a total maxillectomy and primary reconstruction with a combined osteomyocutaneous fibula flap and a custom-made, additively manufactured subperiosteal implant.

The planning process for the patient-specific implant and the associated fibula osteotomies was based on the high-resolution CT scans of the craniofacial region (in DICOM format) to assess the maxillary defect and define the extent of bone removal. Optical impressions of the dental arches and a diagnostic wax-up were also obtained to ensure that the final implant would accommodate a suitable prosthetic rehabilitation, mirroring the patient’s ideal occlusion and aesthetic parameters. In addition to the craniofacial scans, CT angiography of the lower limbs was performed. This provided critical information about the vascular anatomy of the fibula and guided the planning of fibular segment harvest and osteotomies.

The acquired DICOM datasets were forwarded to Sintac Biomedical Engineering (GPI group, Trento, Italy). Using Mimics software (Materialise, Leuven, Belgium), the medical imaging data were segmented and converted into an STL file, generating a high-fidelity three-dimensional model of the maxilla and the relevant anatomical structures. During this phase, any artifacts or inconsistencies in the imaging data were addressed to ensure an accurate virtual representation of the patient’s anatomy.

**Fig. 2:**
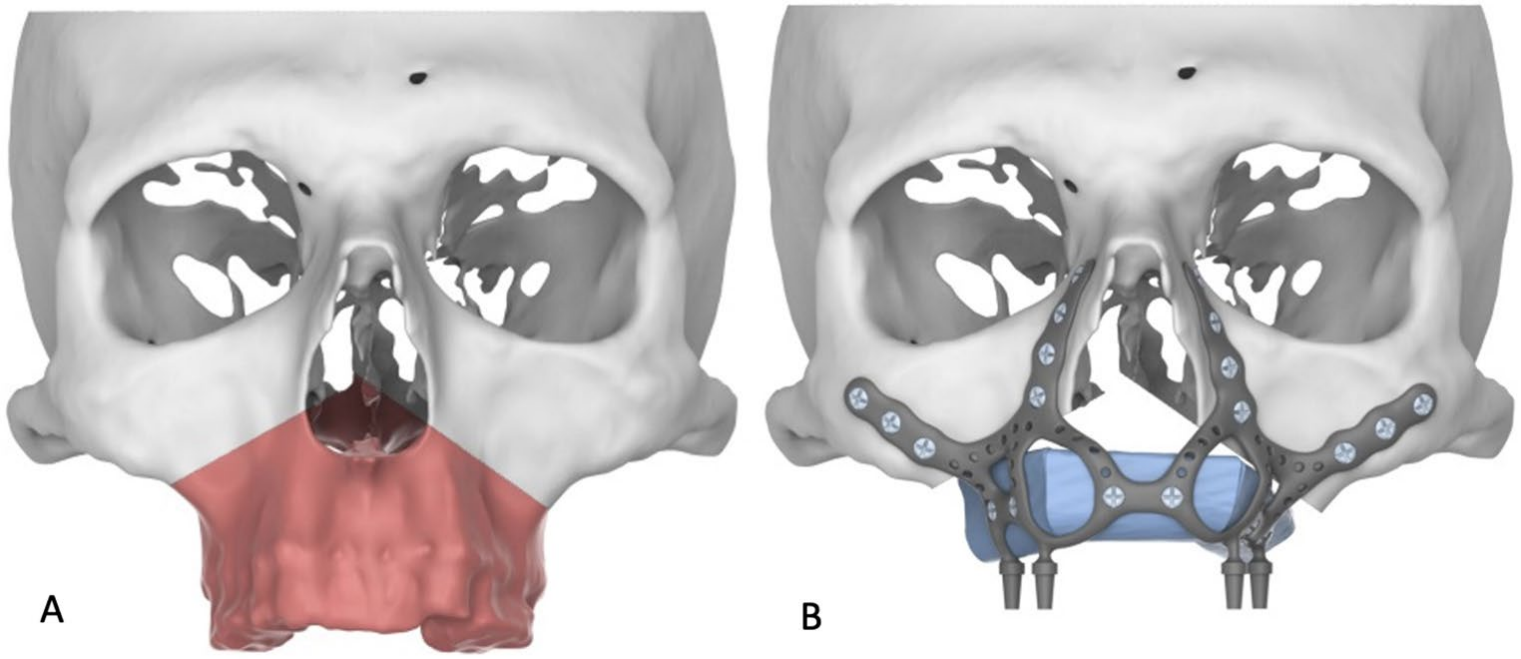
Virtual surgical planning. (**A**) Three-dimensional reconstruction of the midface and maxilla, highlighting in red the planned resection area for the total maxillectomy. (**B**) Design of the patient-specific subperiosteal implant (gray) and fibula bone segments (blue). Note the integrated fixation holes and extended arms to accommodate intraoperative variations

Next, the design of the patient-specific subperiosteal implant proceeded in Geomagic Freeform software (3D Systems Inc., Rock Hill, SC, USA). The implant was virtually adapted to the planned resection margins, factoring in the post-maxillectomy defect that would be created (Fig. 2A). Multiple parameters were considered, including the anticipated positioning and angulation of prosthetic abutments, strategic placement of fixation holes for osteosynthesis screws, and extended arms to accommodate intraoperative variations (Fig. 2B).

Concurrently, the CT angiography of the lower limbs was integrated into the planning. These images guided the optimal harvest site from the fibula and informed the design of patient-specific cutting guides for the bone segments. These guides ensured that the fibula would be segmented precisely as required to fit the newly created maxillary defect. The digital plan defined the length and orientation of the fibular segments and their alignment with the implant framework, enabling a stable and anatomically compatible reconstruction.

Upon completion of the virtual designs, the engineering team presented the comprehensive plan, including the implant, fibula osteotomies, and cutting guide designs, to the surgical team. A collaborative review allowed for iterative refinements, ensuring that the surgical plan was both anatomically accurate and practically feasible. Once approved, the implant was fabricated using direct metal laser sintering (DMLS) technology in a Ti6Al4V titanium alloy. The titanium implant underwent heat treatment to enhance its mechanical properties—improving fracture toughness, thermal stability, and dimensional reliability under load. Selective 3D printing techniques were used to fabricate the fibula cutting guides and anatomical models from high-performance polymers (e.g., polyether ether ketone). The entire cycle from data acquisition to the final sterilized implant and cutting guides took approximately ten days, ensuring no delays in the planned surgery.

**Fig. 3:**
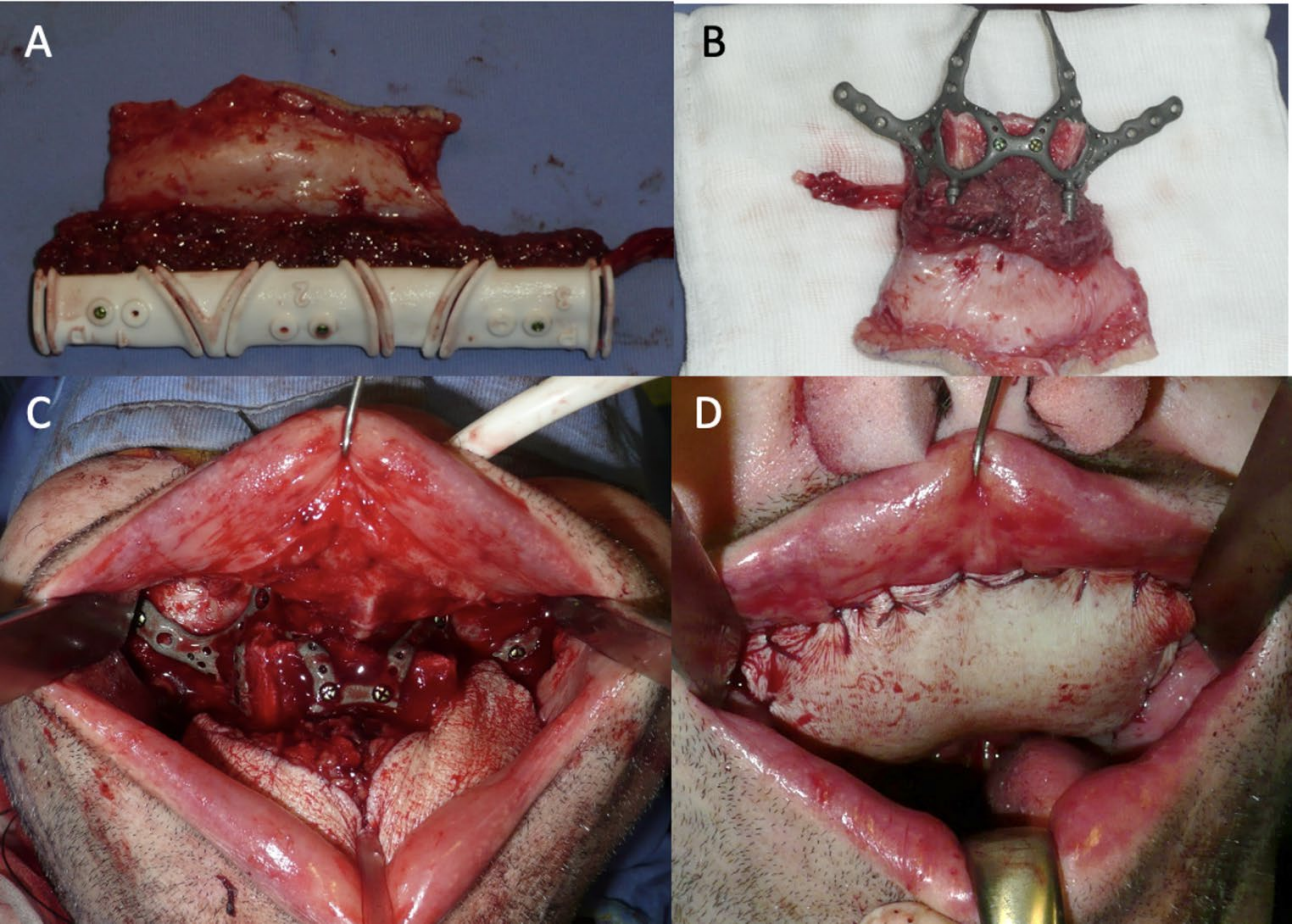
Intraoperative stages of reconstruction. (**A**) The fibula bone segment with the surgical template used to perform the osteotomies. (**B**) The custom-made subperiosteal implant fixed onto the fibula flap prior to inset. (**C**) Intraoral view of the implant and fibular segments rigidly secured with osteosynthesis screws, reconstructing the maxillary defect. (**D**) Immediate postoperative closure showing proper soft tissue adaptation over the reconstructed area

Under general anesthesia, the patient underwent a total maxillectomy with intraoperative frozen section analysis to confirm tumor-free margins. The previously planned fibula osteotomies were executed using the patient-specific cutting guides (Fig. 3A), ensuring precise segment preparation. The custom subperiosteal implant was then positioned to provide a stable foundation for the fibula segments (Fig. 3B), which were rigidly fixed with osteosynthesis screws through the integrated design features of the implant (Fig. 3C). Microvascular anastomosis of the fibular pedicle was performed to the facial artery and vein using end-to-end anastomosis. No vascular grafts were required, as the recipient vessels provided adequate pedicle length to ensure a tension-free anastomosis.

The fibular flap’s soft tissue paddle was contoured to recreate the palatal surface and re-establish the separation between the oral and nasal cavities (Fig. 3D). Closure was achieved without complication, and the patient was transferred to the recovery ward for postoperative care.

Histopathological analysis confirmed a pT4a squamous cell carcinoma, and the patient subsequently underwent adjuvant radiotherapy. Radiotherapy was initiated six weeks postoperatively to allow for optimal soft tissue healing and to minimize peri-implant mucosal irritation. During the radiotherapy period, the transmucosal abutments remained submerged to minimize mucosal irritation and facilitate healing. After completing radiotherapy, the abutments were exposed, and a provisional prosthesis was delivered, restoring the patient’s masticatory function, phonetics, and aesthetics. Six months postoperatively, once the soft tissues had fully matured, a definitive prosthesis was fabricated and loaded.

**Fig. 4:**
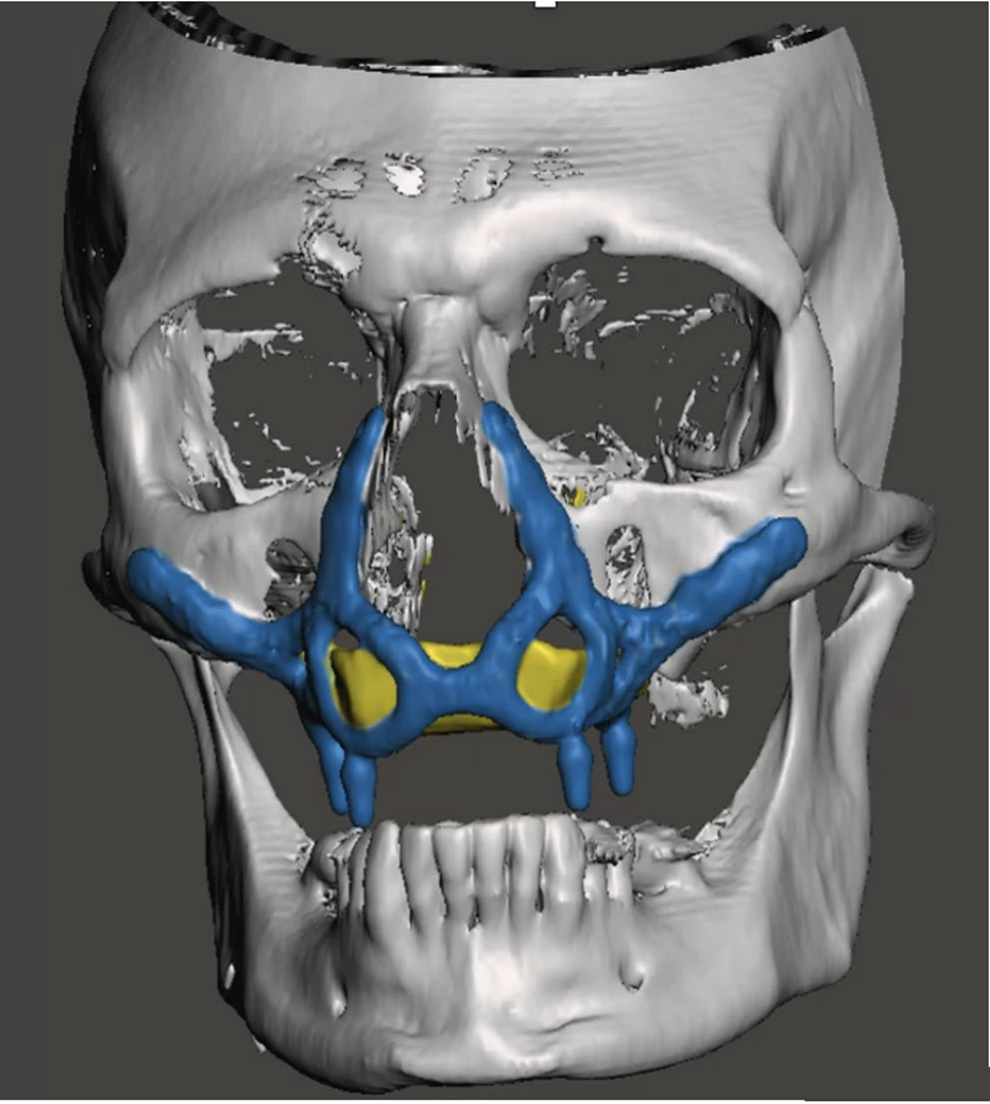
Post-operative 3D CT scan one year after the surgery

**Fig. 5:**
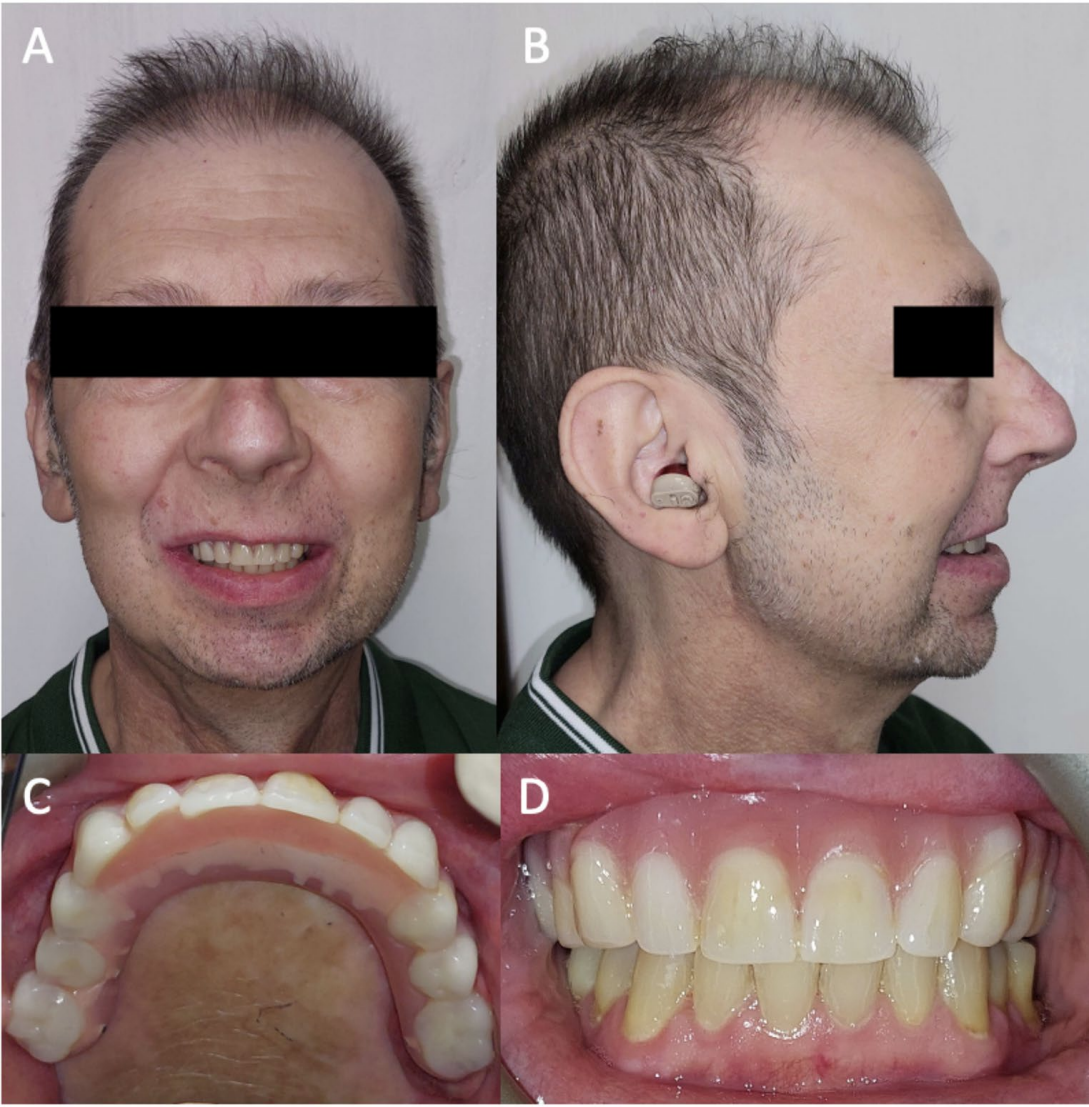
Two-year postoperative results. (**A**) Frontal and (**B**) lateral extraoral views demonstrating symmetrical facial contours, stable soft tissue support, and no external deformities. (**C**) Intraoral view of the definitive prosthesis fully integrated with the reconstructed maxilla, restoring palatal form and occlusal function. (**D**) Frontal intraoral view

At the two-year follow-up, the patient remained alive and free of disease recurrence. Clinical and radiographic evaluations showed stable implant integration (Fig. 4), healthy peri-implant soft tissues, and the absence of any mechanical or biological complications. The prosthetic rehabilitation remained fully functional, contributing to a significant improvement in the patient’s quality of life (Fig. 5).

## Discussion

This case demonstrates the feasibility and potential benefits of employing additively manufactured subperiosteal implants in a primary maxillary reconstruction scenario. While most literature has focused on their use in secondary reconstructions [10–13]—after the anatomy has stabilized and scarring has occurred—this report underscores the possibility of integrating a patient-specific implant simultaneously with tumor resection and free flap reconstruction. By doing so, the patient benefitted from a tailored bony and prosthetic foundation that facilitated early functional and aesthetic rehabilitation.

Compared to endosseous implants, subperiosteal implants offer several advantages in complex maxillary reconstructions. They do not require significant residual bone volume for osseointegration, making them particularly useful in cases of severe maxillary atrophy or after extensive oncologic resections. Their custom design ensures a precise fit and optimal prosthetic positioning, allowing for immediate functional rehabilitation. Moreover, unlike endosseous implants, they do not necessitate secondary surgeries for bone grafting or implant placement within the fibular flap, which is particularly advantageous in patients requiring postoperative radiotherapy. Avoiding additional surgical interventions on the flap reduces the risk of complications such as delayed healing and osteoradionecrosis. However, subperiosteal implants also have some disadvantages, including a higher risk of peri-implantitis due to their close soft tissue interface, potential soft tissue irritation from exposed components, and the complexity of fabrication, which requires meticulous digital planning and close collaboration between surgeons and biomedical engineers. Unlike endosseous implants, which rely on direct bone integration, subperiosteal implants depend on mechanical fixation to the residual skeletal structures, which may lead to long-term stability concerns if not properly designed and placed.

One key advantage of incorporating custom-made implants in primary settings is the capacity to address functional and anatomical requirements before tissue contraction and scar formation. In contrast to secondary reconstructions, where the topography may be altered and less predictable, primary integration allows for a more direct and immediate adaptation of the implant to the patient’s original anatomy (or closely estimated defect), thereby enhancing prosthetic outcomes and potentially reducing the number of secondary surgeries. The implant not only provides a stable scaffold for the fibula flap but also positions prosthetic abutments in optimal locations to support a future prosthesis. This strategic prosthetic-driven design can streamline the rehabilitation timeline and reduce patient morbidity.

The prompt availability of patient-specific implants is crucial for integration into primary oncologic workflows. In this case, the production cycle was accomplished in approximately ten days, allowing surgery to proceed without delay. This is a noteworthy improvement over past workflows, where extended fabrication times often limited patient-specific implant applications primarily to delayed reconstructions [11].

Another significant advantage of this approach lies in its adaptability. Preoperative planning accounted for potential intraoperative variations, including the possibility of more extensive bone resection than initially anticipated. By incorporating extended arms and additional fixation holes into the implant design, surgical teams gain flexibility during the procedure to maintain stable fixation even if the final defect is larger than planned.

However, as with any innovative technique, certain considerations must be addressed. In patients requiring adjuvant radiotherapy, the presence of transmucosal abutments can pose a risk of mucosal toxicity and delayed healing [11, 12]. In this report, the abutments remained submerged during radiotherapy and be uncovered only after treatment completion, mitigating these potential complications.

This case also highlights that, when executed with careful planning and a multidisciplinary approach, primary application of a custom subperiosteal implant can achieve stable and predictable outcomes. Over the two-year follow-up, no implant-related complications were noted, and the patient remained free of disease. Such findings should be confirmed on large series but align with previous reports in secondary reconstructions [10–13], suggesting that the early integration of patient-specific implants need not compromise oncologic safety or prosthetic success.

Nonetheless, this is a single case experience. Larger prospective studies and longer follow-up periods are needed to draw more definitive conclusions regarding long-term implant survival, peri-implant tissue stability, and the broader cost-effectiveness of this approach. In addition, the complexity of digital workflows—requiring meticulous imaging, planning, and fabrication—calls for seamless collaboration among surgeons, biomedical engineers, prosthodontists, and radiologists.

In conclusion, this case adds to the growing body of evidence that additively manufactured subperiosteal implants can play a valuable role in primary maxillary reconstruction. By offering a stable, custom-tailored platform for both bone and prosthetic support, these implants have the potential to open new avenues for improving patient outcomes, expediting rehabilitation, and expanding treatment possibilities in complex oncologic scenarios.

## Data Availability

No datasets were generated or analysed during the current study.
